# Fluorination of silyl prosthetic groups by fluorine mediated silyl ether linker cleavage: a concept study with conditions applicable in radiofluorination

**DOI:** 10.1186/s41181-022-00167-y

**Published:** 2022-06-25

**Authors:** Carsten Sven Kramer, Luca Greiner, Klaus Kopka, Martin Schäfer

**Affiliations:** 1 Radiopharmaceutical Chemistry, German Cancer Research Center (DKFZ), Im Neuenheimer Feld 223, 69120 Heidelberg, Germany; 2 Institute of Radiopharmaceutical Cancer Research, Helmholtz-Zentrum Dresden-Rossendorf (HZDR) e. v., Bautzner Landstrasse 400, 01328 Dresden, Germany; 3 Fakultät Chemie Und Lebensmittelchemie, Technische Universität Dresden, Mommsenstraße 4, 01062 Dresden, Germany

**Keywords:** Fluorine-18, PET, SiFA, Solid phase synthesis, Radiofluorination, Solid support, Detagging, Silyl fluorides

## Abstract

**Background:**

Positron emission tomography (PET) is a powerful tool in medical imaging, especially in combination with the PET radionuclide fluorine-18 that possesses optimal characteristics. For labelling of biomolecules and low-molecular weight tracers, fluorine-18 can be covalently bound to silicon by either nucleophilic replacements of leaving groups (like ethers) or by isotope exchange of fluorine-19. While nucleophilic substitutions require additional purification steps for the removal of contaminants, isotope exchange with fluorine-18 results in low molar activity. Both challenges can be addressed with a detagging-fluorination of an immobilized silyl ether motif.

**Results:**

By overcoming the susceptibility towards hydrolysis, optimized detagging conditions (improved reaction time, fluorination reagent, linker, and resin) could afford the highly sterically hindered silyl fluoride motifs, that are commonly applied in radiochemistry in small and semipreparative scales. The described reaction conditions with fluorine-19 are transferrable to conditions with [^18^F]fluoride and silyl fluorides were obtained after approx. 10 min reaction time and in high-purity after mechanical filtration.

**Conclusions:**

We present a proof-of-concept study for a detagging-fluorination of two silyl ethers that are bound to an optimized amino alcohol resin. We show with our model substrate that our solid-phase linker combination is capable of yielding the desired silicon fluoride in amounts sufficient for biological studies in animals or humans under standard fluorination conditions that may also be transferred to a radiolabelling setting. In conclusion, our presented approach could optimize the molar activity and simplify the preparation of radiofluorinated silyl fluorides.

## Background

Over the past decades, PET has found its way in diagnostic medicine as a powerful non-invasive imaging tool to study physiological functions (such as cardiac PET), to assist in the diagnosis of diseases such as cancer or inflammation, and to assess therapy response (Vaquero and Kinahan 2015). The PET nuclide fluorine-18 has as a diagnostic radionuclide very beneficial properties (Cole et al. 2014): the relative long half-life (109.8 min), the low emission energy of emitted positrons (E_max_ = 633 keV), the high positron emission probability (97%), the absence of additional γ-lines, and its good availability due its the simple production path (^18^O(p,n)^18^F route) makes fluorine-18 to an nearly ideal non-metal radioisotope for PET applications.

In the clinical setting, fluorine-18 is commonly introduced covalently in the n.c.a. (non-carrier-added) form by applying simple nucleophilic displacements or isotope exchange reactions. In principal, due to the low concentrations of a radionuclide, the kinetics of labelling reactions are usually characterized by their (pseudo)first order and thus demand a sufficient amount of the corresponding synthon to achieve an acceptable reaction rate (Holland 2018). Usually, this excess of labeling synthon must be separated by time-consuming purifications and subsequent reformulation steps. Consequently, we propose a solid phase method in combination with a fluorophilic linker.

**Fig. 1:**
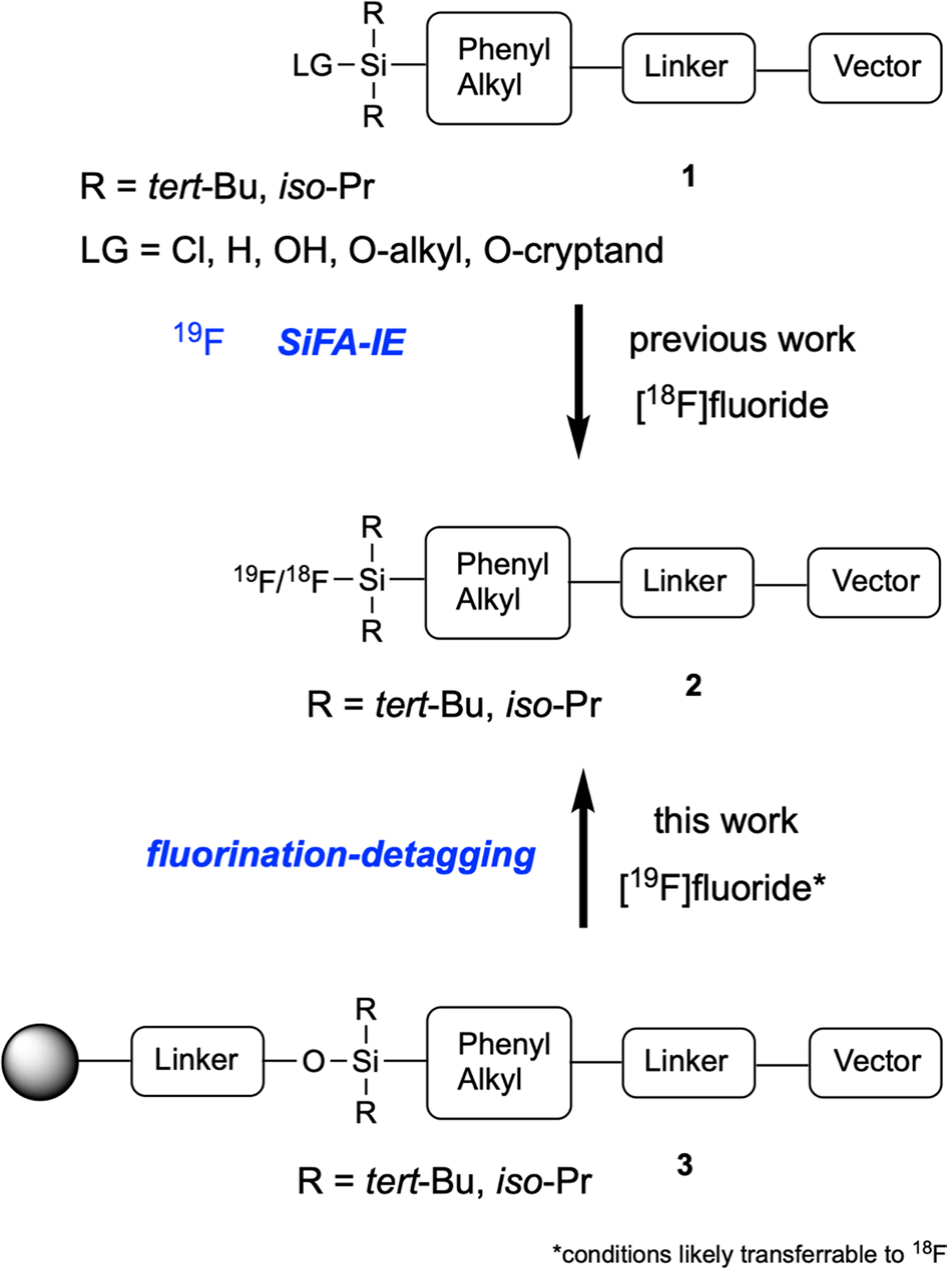
Methods for the radiofluorination of silicon-based fluoride acceptors (SiFA). Leaving groups (LG) can be exchanged with (non)radioactive fluoride; a special case is the isotope exchange of non-radioactive fluoride with its radioactive counterpart (SiFA-IE); References for the replacement of different LGs with fluorine can be found in (Bernard-Gauthier et al. 2014)

Whereas metals need to be complexed with chelators to molecules of interest, fluorine can undergo stable bond formations with carbon or heteroatoms such as boron, aluminum or silicon. Using silicon for fluorination is highly attractive since a silicon-bearing building block can be introduced early in the synthetic route of the precursor molecule and conveniently carried over to the final product (in contrast to boron or aluminum). Also, no chelators – that might alter the hydrophilicity dramatically – or highly reactive precursors (like used for C-F formations) are necessary for silyl fluoride formation. Fluorination of silicon occurs in mild conditions with readily available precursors (Fig. 1) (Bernard-Gauthier et al. 2014).

The silicon fluorine bond is in general prone to hydrolysis, thus it is necessary to decorate the silicon atom with bulky organic residues (such as *tert*-butyl or *iso*-propyl groups, like in **1**, Fig. 1) to shield it from an attack of water and other nucleophiles (Bernard-Gauthier et al. 2014). The leaving group also plays an important role, as it determines the kinetics, the conditions of the replacement with fluorine, and in addition the generation of potential contaminants (e. g. alcohols when using silyl ethers as precursors). Figure 1 compromises examples of leaving groups, that can be substituted with [^18^F]fluoride. A special case is the SiFA-IE (silicon fluoride acceptor isotope exchange), an elegant approach for mild radiolabelling, since the chemical structures of precursor and the radiolabelled product are identical (^19^F-**1** to ^18^F-**2**) (Bernard-Gauthier et al. 2014). In theory, the only contaminants can be the non-radioactive fluorinated precursor and the fluorination reagents (widely used is a mixture of base, cryptand and a fluoride salt). Despite the clear advantages, radiolabelling using the SiFA-IE offers the product mostly with low specific activity since radioactive and non-radioactive fluoride ions compete at the silicon.

In this proof-of-concept study we want to show that bulky silicon fluorides (that appear water-stable due to kinetically inhibited hydrolysis) can be synthesized by a fluorine mediated cleavage (detagging-fluorination) of an immobilized silicon ether precursor (**3** to **2**, Fig. 1). We assume that the application of an immobilized (bio)vector molecule bridged via a silyl ether with the solid support could overcome several crucial problems. To stress the most important, due to the selectivity of fluoride to silicon, the labelled (bio)vector is supposed to be the only cleaved species.

To best of our knowledge, just a few reactions are known in which a fluorination reaction accomplishes the cleavage from solid support: Luthra and co-workers presented the synthesis of [^18^F]FDOPA by a detagging-fluorination of a stannane-linked DOPA-precursor with an electrophilic [^18^F]fluorine source (Luthra et al. 2006). Using non-radioactive (Demizu et al. 2012) and radioactive (Lutra et al. 2002) nucleophilic fluorine sources, FDG precursors could be cleaved from polymer-bound sulfonic esters. Keng and co-workers also used a polymer-bound sulfonic ester to cleave a test substrate in a microfluidic chip under radioactive conditions (Ismail et al. 2012). Furthermore, the Bräse group used Olah’s reagent to introduce a geminal difluoro motif by detagging from a solid support bound dithiane linker system (Wiehn et al. 2008). Nevertheless, all those methods enable the formation of a C-F but not of a Si–F bond. Furthermore, if such a detagging silyl fluorination would meet the demands compulsory for the synthesis of radiotracers (reagents also applicable in radiosynthesis like KF/cryptand-mixtures, short reaction times, minimal post-reaction purification and reformulation steps), the method would also allow radiolabelling concurrent with cleavage from solid support (**3** to ^18^F-**2**) and enable access to high molar activities of SiFA-tagged (bio)vectors. Therefore, we assume that the molar activity of the labelled molecule and the [^18^F]fluoride would be equivalent and a subsequent elaborate separation of the precursor and the labelled product would be gratuitous as the immobilized labelling synthon can be easily removed by mechanical filtration. Separation of polar additives or unbound [^18^F]fluoride should be easily achieved by solid-phase-extraction (SPE).

## Methods

All **chemicals and solvents** were purchased from Sigma-Aldrich, abcr GmbH, Acros Organics and were used as received without further purification. The resins were purchased from Merck Millipore. Deuterated solvents were used from Deutero GmbH. **NMR spectra** were recorded at room temperature on a Bruker Avance III (400 MHz) spectrometer. Chemical shifts are reported in δ units relative to d-chloroform (δH = 7.26; δC = 77.2). Analyses followed first order and the following abbreviations were used throughout: s = singlet, d = doublet, t = triplet, dd = doublet of doublet etc., m = multiplet. Coupling constants (*J*) are given in Hz and refer to H,H- or Si,F-couplings. **Mass spectra (MS)** were determined in the Institute for Organic Chemistry of the University of Heidelberg under the direction of Dr. Jürgen Gross. High-resolution mass spectra (HRMS) were recorded with a JEOL JMS-700 spectrometer. The molecule ions are reported as mass to charge (m/z) relation. **Analytical HPLCs** were performed on an Agilent 1100 series equipped with a G1322A degasser, G1311A-QuatPump, G1313A-ALS-autosampler, G1315B-DAD, and a Chromotlith® Performance column (RP-18e, 100–4.6 mm, Merck KGaA); solvent A = water with 0.1% TFA and solvent B = acetonitrile with 0.1% TFA; pressure = 60 bar; flow rate: 2 mL/min; gradient sequence: min = 0 (A: 90%, B: 10%) to min = 10 (A: 0%, B:90%); detection wavelengths: 220 nm and 254 nm).

### Synthesis of silyl fluoride 7 (analytical standard):

In a falcon tube TBDPSCl (203 mg, 0.738 mmol) was placed and dissolved in dry THF (6.0 mL). Then the solution was treated with Olah’s reagent (22.3 mg, 1.11 mmol, 1.5 equiv.) at 0 °C. The reaction mixture was allowed to come to room temperature while stirring o.n.. Then sat. sodium bicarbonate solution was *carefully* added to the mixture (*attention*: gas is evolving). The mixture was diluted by addition of water and DCM. The phases were separated, and the water phase was extracted three times with DCM. The collected organic phases were dried with brine. After drying with sodium sulfate, filtration and evaporation of solvents, 190.1 mg (0.738 mmol, quant. yield) of a slight yellow oil were obtained.

^**1**^**H NMR** (400 MHz, CDCl_3_) δ = 7.75 – 7.68 (m, 4 H), 7.50 – 7.36 (m, 6 H), 1.10 (d, *J* = 1.1, 9 H). ^**19**^**F NMR** (376 MHz, CDCl_3_) δ = − 185.26 (96% as s, 4% as d, *J* = 290 Hz). ^**29**^**Si NMR** (79 MHz, CDCl_3_) δ = 3.55 (d, *J* = 290 Hz). **HR MS** (ESI) calc. for C_16_H_19_FSi: 258.12323, found: 258.12346. Retention time of silyl fluoride **7**: 7.3 min.

### Synthesis of silyl fluoride 12 (analytical standard):

In a falcon tube chloro(di *iso*propyl)(2-phenylethyl)silane (55 mg, 0.216 mmol) was dissolved in THF (1.0 mL). Under ice bath cooling, the solution was treated with HF (48 wt% in water, 100 µL). The reaction mixture was allowed to come to room temperature while stirring o.n.. Then sat. sodium bicarbonate solution was *carefully* added to the mixture (*attention*: gas is evolving). The mixture was diluted by addition of water and diethyl ether. The phases were separated, and the water phase was extracted three times with diethyl ether. The collected organic phases were dried with sodium sulfate and filtered off. The solvents were carefully evaporated at rotavap (at 40 °C rotavap bath temperature, 15 mbar vacuum) and 49.4 mg (0.207 mmol, 96% yield) of a slight yellow oil were obtained.

^**1**^**H NMR** (400 MHz, CDCl_3_) δ = 7.32 – 7.27 (m, 2 H), 7.24 – 7.16 (m, 3 H), 2.80 – 2.70 (m, 2 H), 1.15 – 1.03 (m, 16 H). ^**13**^**C NMR** (101 MHz, CDCl_3_) δ = 144.85, 128.57, 127.82, 125.90, 28.93, 28.92, 17.02, 17.00, 16.98, 13.03, 12.90, 12.57, 12.44. ^**19**^**F NMR** (376 MHz, CDCl_3_) δ = − 184.86. (93% as s, 7% as d, *J* = 296 Hz). **HR MS** (ESI) calc. for C_14_H_23_FSi: 238.15476, found: 238.15432. Retention time of silyl fluoride **12**: 7.7 min.

### Synthesis of the amino alcohol silyl ether 6 (immobilized on 2-(4-bromomethylphenoxy)ethyl polystyrene HL resin)

The 2-(4-bromomethylphenoxy)ethyl polystyrene HL resin **4** (830 mg, 1.23 mmol/g, 1.0 mmol) was pre-swelled for 60 min in 10 mL dry DCM. Then 2-(2-aminoethoxy) ethanol (1.58 g, 15 mmol, 15 equiv.) was added to the resin. The reaction mixture was stirred over o.n. at room temperature. The resin was washed three times with DMF, three times with DCM, and three times with *n*-hexane. Then the resin was dried *in vacuo*. In this way 734 mg of the resin amino alcohol modified resin were obtained. The resin (734 mg) was pre-swelled for 1 h with 3 mL dry DCM. Then dry DMF (10 mL), DMAP (551 mg, 4.5 mmol, 4.5 equiv.), DIPEA (1.22 mL, 12.4 mmol, 12.4 equiv.) and TBDPSCl (3.71 g, 13.5 mmol, 13.5 equiv.) were added. The reaction mixture was stirred for o.n. at room temperature. The resin was washed three times with DMF, three times with DCM, and three times with *n*-hexane. Then the resin was dried *in vacuo*. 820 mg of the silyl ether resin **6** were obtained.

### Synthesis of the amino alcohol silyl ether 11 (immobilized on 2-(4-bromomethylphenoxy)ethyl polystyrene HL resin)

The amino alcohol modified resin (50 mg, synthesized from resin **4** with a substitution of 1.23 mmol/g) was pre-swelled for 1 h with 1 mL dry DMF. Then DMAP (37 mg, 0.302 mmol), DIPEA (810 μL, 0.601 mmol), and chloro(di *iso*propyl)(2-phenylethyl)silane (299 mg, 308 μL, 0.97 g/mL, 1.17 mmol) were added. The reaction mixture was stirred for 1 d at rt. The resin was washed three times with DMF, three times with DCM, and three times with *n*-hexane. Then the solid phase was dried *in vacuo* to yield 45 mg of silyl ether resin **11**.

### General procedure for the detagging-fluorination on analytical scale

The resin (10–15 mg) was placed in a reaction syringe (for solid-phase peptide synthesis, equipped a HDPE-filter) and washed four times with dry THF under argon. Best practice for the washing step: all steps need to be done under argon. A sufficient amount of dry THF was filled in a heatgun-dried flask that is equipped with a sleeve stopper and an argon balloon. The reaction syringe, loaded with the resin, was equipped with a long needle and dry THF was filled in the syringe. The syringe gently was gently shaken, then the THF was thrown away in a second ‘waste’ flask, that was equipped with a sleeve stopper and an argon balloon. Then fresh dry THF was filled in the syringe, the syringe was shaken, and the solvent was discarded. In total, the solid phase should be washed/dried for 3–4 times, at the end of the washing step the syringe should only contain the solid phase and no solvent). In another flame-dried flask, that was equipped with a sleeve stopper and filled with argon, Kryptofix®222 (75 mg), potassium carbonate (50 mg) and KF (25 mg) was placed. Then 1 mL of dry THF was added in the flask and the flask was gently shaken (nota bene: not all material dissolves). The dissolved material was taken up in the reaction syringe (loaded with the pre-dried solid phase) and the syringe was closed with a stopper. Then the mixture was shaken by hand for 8 min. The mixture was filtered (over the reaction syringe filter), collected in a HPLC vial and analyzed.

Retention time of silyl fluoride **7**: 7.3 min.

Retention time of silyl fluoride **12**: 7.7 min.

### Detagging-fluorination procedure of 7 on semi-quantitative scale

Silyl ether resin **6** (123 mg, synthesized from resin **4** with a substitution of 1.23 mmol/g) was placed in a reaction syringe and washed multiple times with dry solvents (e. g. THF or diethyl ether, see method above). Kryptofix®222 (250 mg), potassium carbonate (276 mg) and KF (116 mg) were quickly added and the syringe was flushed with argon. Then 1.5 mL dry THF was taken up in the reaction syringe and the syringe was closed with a stopper. The syringe was shaken for 8 min. The supernatant was collected, and the solid phase was washed multiple times with THF. The unified organic solvents were diluted with DCM. Then 0.1 N (aqu.) HCl was added and the aq. phase was extracted multiple times with DCM. The collected organic solvents were dried with sodium sulfate, filtered and the solvents were evaporated *in vacuo* to give 22 mg (56% yield) of silyl fluoride **7** as a colorless oil. HPLC–DAD, MS und NMR (H, Si, F) data proved identity and purity of the product and were in accordance with the data acquired from the material prepared as standard.

## Results

**Fig. 2:**
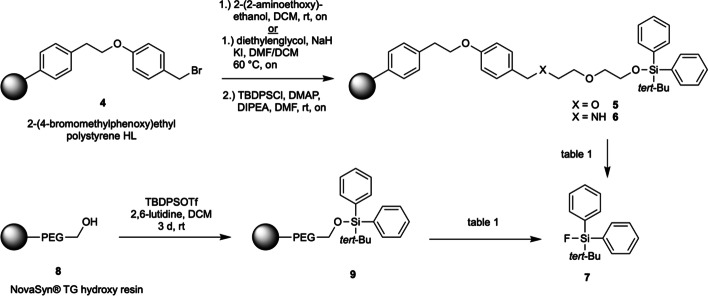
Detagging conditions of silyl ethers **5**, **6**, and **9**

In a first attempt, the 2-(4-bromomethylphenoxy)ethyl polystyrene HL resin was modified with a diethylene glycol spacer (Fig. 2 and Table 1) (Ngu and Patel 1997). The so obtained immobilized alcohol was converted to the *tert*-butyl diphenyl silyl (TBDPS) ether **5**. Previous studies showed that the cleaving product TBDPSF possesses reasonable hydrolytic stability in water (close to 100% stability in water or phosphate buffered saline over 5 h at 45 °C (Choudhry et al. 2006)), therefore we assumed that the TBDPS silyl ether **5** could be a suitable model substrate to test the nucleophilic detagging with fluoride (in addition, the di(*tert*-butyl)phenyl silyl analogue is not commercially available as its chloride or triflate). With the diethylene glycol-linked TBDPS silyl ether **5** in hand, we tested different cleaving conditions. The reactions were performed on an analytical scale (10–15 mg resin) in a polypropylene peptide synthesis reactor, and the solvent phase was subsequently analyzed by HPLC. HPLC standards of fluoride **7** and the corresponding silanol were synthesized by treatment of TBDPSCl with Olah’s reagent, or by hydrolysis of TBDPSOTf (only analytical scale), respectively. Treating diethylene glycol modified resin **5** with standard fluorination conditions (KF, K_2_CO_3_, Kryptofix®222), the desired fluoride **7** could be detected by HPLC as traces along with the silanol product as the main peak (Entry 1). Even when using very dry solvents and extra washing of the resin, silanol formation was unavoidable. Subsequently, we tested the commercially available PEGylated NovaSyn® TG hydroxy resin **8** to avoid any contamination of the solid phase that could lead to silanol formation. A first attempt led to pure silanol formation (Entry 2) and even with azeotropically dried fluorination reagents and higher reaction temperature with longer reaction time, no silyl fluoride **7** could be detected (Entry 3); also, attenuation of reaction conditions didn’t generate the desired fluorination product (Entry 4).

**Table 1:** Detagging conditions of silyl ethers **5**, **6**, and **9**

Entry	Linker	Cleavage conditions	Result (HPLC analysis)
1	Diethylenglycol	KF, K_2_CO_3_, Kryptofix®222, rt, THF, 10 min	Silanol (major) Fluoride **7** (minor)
2	NovaSyn® TG hydroxy resin	KF, K_2_CO_3_, Kryptofix®222, rt, THF, 10 min	Silanol (pure)
3	PEG linker NovaSyn® TG hydroxy resin	KF, K_2_CO_3_, Kryptofix®222 (note: pre-drying of fluorination reagents by three times co-evaporation with ACN at 120 °C under vacuum), then reaction at 55 °C, THF, 30 min	Silanol (pure)
4	PEG linker NovaSyn® TG hydroxy resin	Pre-drying of solid phase, KF, K_2_CO_3_, Kryptofix®222, rt, 8 min	Silanol (pure)
5	2-(2-aminoethoxy)ethanol	Pre-drying of solid phase, KF, K_2_CO_3_, Kryptofix®222, rt, 7 min	Fluoride **7** (pure)
6	2-(2-aminoethoxy)ethanol	CsF, K_2_CO_3_, Kryptofix®222 (all reagents pre-dried), rt, ACN, 30 min	Silanol (major) Fluoride **7** (traces)
7	2-(2-aminoethoxy)ethanol	KF, K_2_CO_3_, 18-crown-6, THF, rt, 10 min	Fluoride **7** (major) Silanol (minor)
8	2-(2-aminoethoxy)ethanol	TBAF, THF, rt, 2 h	Silanol (pure)
9	2-(2-aminoethoxy)ethanol	Olah’s reagent, THF, 0 °C, 8 h	Fluoride **7** (pure)
10	2-(2-aminoethoxy)ethanol	Pre-drying of solid phase, KF, K_2_CO_3_, Kryptofix®222, rt, 8 min	Fluoride **7** (pure) (semiquantitative scale) (56% overall yield over 3 steps)

Modification of 2-(4-bromomethylphenoxy)ethyl polystyrene HL resin **4** with 2-(2-aminoethoxy)ethanol and subsequent formation of the silyl ether led to substrate **6** to test the cleavage reaction. Standard fluorination conditions paired with a short reaction time provided a clear fluoride signal in the HPLC chromatogram (Entry 5). With a longer time, a silanol peak became visible due to the instability of the silyl fluoride in basic media. Also, other fluorination conditions were tested on the aforementioned linker-resin combination: Exchange of KF with CsF provided only fluoride traces (Entry 6) whereas utilization of 18-crown-6 instead of Kryptofix®222 led to dominant fluoride formation along with some silanol byproduct (Entry 7). When subjected to TBAF, the hydrolysis product was detected solely (Entry 8). Use of Olah’s reagent led to a clear fluoride formation (Entry 9), proving to be a suitable system for silyl fluoride synthesis (that can be later PET-activated by isotope exchange)), but it will be unlikely to transfer this fluorination condition into a direct radiofluorination method. Nevertheless, starting from this observation, radiofluorination conditions using the recently published iron-trapped [^18^F]F species might be applicable (Verhoog et al. 2019).

Satisfyingly, the fluorination-untagging reaction with KF, K_2_CO_3_, Kryptofix®222, conditions that are most likely applicable for radiosynthesis, worked also on semi-preparative scale: by using 123 mg of resin **6**, 22 mg of TBDPSF (**7**) could be obtained after a short extraction; HPLC, NMR (H, F, Si), MS confirmed its identity and high purity. Since TBDPSCl was used in excess it would be unreasonable to give an overall yield describing the conversion from the silyl chloride to the fluoride, calculating from fully loaded resins (approx. 1.23 mmol/g), the detagging reaction to **7** would proceed in 56% (overall-)yield (over three steps), making the detagging-fluorination very mass efficient.

**Fig. 3:**
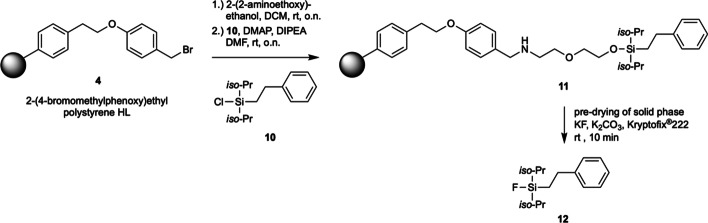
Detagging of di-*iso*propyl silyl fluoride **12** from immobilized amino alcohol silyl ether **11**

Encouraged by positive findings, we examined whether the fluorination works also efficiently with silyl fluoride moieties commonly used as radioactive prosthetic groups. The di-*iso*propyl-silyl-phenyl-moiety is more stable to hydrolysis but also more challenging to fluorinate due to its bulkyness. Silyl ether **12**, prepared from the immobilized amino alcohol and the di-*iso*popyl silylchoride **10**, was examined in the detagging-fluorination with the optimized conditions (Fig. 3). To our delight, we found only the fluorinated product after 10 min reaction time and absence of silanol product (standard of **12** was prepared from chloride **10** with Olah’s reagent, acidic hydrolysis of **10** yielded the silanol, respectively). It has to be mentioned that the di-*iso*propyl silyl fluoride was found to be stable for at least 3 d in the HPLC vial whereas silyl fluoride **7** was prone to hydrolysis.

## Discussion

In contrast to PEGylated resins **5** and **9**, amino alcohol modified resins **6** and **11** underwent the detagging-fluorination under mild conditions with reagents that are commonly used in radiofluorination. Not only the fluorination reagents are suitable for radiosynthesis, reaction times for the fluorination of substrates **6** and **11** are below 10 min and the obtained products are showing sufficient purity. On immobilized resin **6** we demonstrated that the detagging is also working on a semiquantitative scale. All these features should allow a time efficient synthesis of ^18^F-labelled molecules with high activity yields.

Reasons why the amino alcohol modified resins are leading to fluoride formation whereas the PEGylated resins gave mostly only the silanol, are hypothetical, but assumingly the PEG-linkers could be prone to water retention and therefore accelerate the hydrolysis of the silyl fluorides. If the amino alcohol modified resin **6** wasn’t extensively dried before use, silanol formation was also detectable. The basic amino alcohol could also interfere with the silicon and facilitate the detagging-fluorination process; it is already known that diminishing silicon Lewis acidity contributes to higher stability of the silyl fluorine (Bernard-Gauthier et al. 2014). More studies would be needed here to examine reaction kinetics of the fluorination with nitrogens in different distances and basicity to the silicon center.

## Conclusion

Herein we present a proof-of-concept study for a detagging-fluorination of two silyl ethers that are bound to an optimized amino alcohol resin. We could show with our model substrate that our solid-phase linker combination is capable of yielding the desired silicon fluoride in amounts sufficient for biological studies in animal or humans under standard fluorination conditions that can also be transferred to a radioactive setting. We also demonstrated that the highly congested di-*iso*propyl-Si motif in **11** can be fluorinated with this system. Moreover, the short reaction times are ideally suited for a radiofluorination-untagging process and detagging from solid phase allows to synthesize silicon fluorides in high purity and spares a time-demanding purification step. In comparison to the SiFA isotope exchange, we see an opportunity to also increase the molar activity. Finally, due to the chemical stability of the silyl ether function it should be possible to build an entire SiFA-linked peptide on solid phase and cleave it with simultaneous radiolabelling. Taken together, we conclude a promising potential by using the presented approach as an alternative to classical radiofluorinations in terms of an optimization of the molar activity and straightforwardness.

## Data Availability

All data generated or analysed during this study are included in this published article.

## Abbreviations

ACN: Acetonitrile
DAD: Diode-array detection
DCM: Dichloromethane
DIPEA: *N*,*N*-Diisopropylethylamine
DMAP: 4-(Dimethylamino)pyridine
DMF: *N,N*-Dimethylformamide
DOPA: 3,4-Dihydroxyphenylalanine
ESI: Electrospray ionization
HDPE: High density polyethylene
HPLC: High performance liquid chromatography
LG: Leaving group
(HR)MS: (High resolution) mass spectroscopy
n.c.a.: Non-carrier-added
NMR: Nuclear magnetic resonance
PEG: Polyethylene glycol
PET: Positron emission tomography
SiFA: Silicon-fluoride acceptor
SiFA-IE: Silicon-fluoride acceptor isotope exchange
SPE: Solid-phase-extraction
THF: Tetrahydrofurane
TBDPS: *tert*-Butyldiphenylsilyl
TFA: Trifluoroacetic acid
